# Mechanism of valproic acid-induced hepatic steatosis via enhancing NRF2-FATP2-mediated fatty acid uptake

**DOI:** 10.7150/thno.108593

**Published:** 2025-04-13

**Authors:** Xiaoliang He, Rui Yuan, Ying Chen, Wenni Huang, Zihao Xu, Bixia Wang, Changhui Liu, Tianqin Xiong

**Affiliations:** 1 School of Pharmaceutical Sciences, Guangzhou University of Chinese Medicine, Guangzhou 510006, China.; 2 State Key Laboratory of Traditional Chinese Medicine Syndrome, School of Pharmaceutical Sciences, Guangzhou University of Chinese Medicine, Guangzhou 510006, China.; 3 Key Laboratory of Chronic Disease Prevention and Control of Traditional Chinese Medicine of Guangdong Higher Education Institutes, Guangzhou University of Chinese Medicine, KLGHEI (2024KSYS024), Guangzhou 510006, China.

**Keywords:** valproic acid, hepatic steatosis, nuclear factor E2-related factor 2, fatty acid transport protein 2, fatty acid uptake

## Abstract

**Rationale:** Valproic acid (VPA), a first-line antiepileptic drug, can induce life-threatening hepatic steatosis with prolonged use; however, the underlying mechanisms remain inadequately elucidated. Nuclear factor E2-related factor 2 (NRF2) is a hepatoprotective factor that maintains redox homeostasis; however, increased levels have been observed in VPA-induced hepatic steatosis. Therefore, the present study aimed to investigate the function of NRF2 in VPA-triggered hepatic steatosis.

**Methods:** NRF2 overexpression mice, NRF2 knockout mice, and fatty acid transport protein 2 (FATP2) knockout mice were constructed using adeno-associated virus, homologous recombination, and CRISPR/Cas9 technology, respectively. The mice were then treated with or without oral VPA to induce hepatic steatosis.

**Results:** NRF2 nuclear expression was positively correlated with triglyceride levels in VPA-induced hepatic steatosis. NRF2 overexpression exacerbated VPA-triggered inflammation and steatosis, whereas NRF2 knockout alleviated the effects. Chromatin immunoprecipitation and dual-luciferase reporter gene assay confirmed that FATP2 is a target gene of NRF2. NRF2 exacerbated VPA-induced hepatic steatosis dependent on FATP2. VPA bound to Cys288 and Arg415 of Kelch-like ECH-associated protein 1 (KEAP1), leading to its autophagic degradation and subsequent nuclear translocation of NRF2.

**Conclusions:** Our results revealed a mechanism that VPA binds to specific KEAP1 sites, promoting its degradation and disrupting the KEAP1-NRF2 complex, thereby facilitating NRF2 nuclear translocation. Subsequently, NRF2 activates FATP2 transcription, enhancing fatty acid uptake and contributing to hepatic steatosis. Our findings suggest that inhibiting the NRF2-FATP2 axis could improve VPA-induced hepatic steatosis, offering promising insights into managing drug-induced fatty liver disease.

## Introduction

Valproic acid (VPA) is a first-line treatment for epilepsy and is widely used for managing bipolar disorder and migraine 1, 2. However, prolonged VPA administration is linked to a heightened risk of hepatic steatosis 3. This VPA-induced steatosis typically manifests as microvesicular steatosis, which is strongly associated with severe liver injury 4 and advanced fibrosis 5. Furthermore, the Food and Drug Administration has issued warnings highlighting the life-threatening risk of severe hepatotoxicity associated with VPA use.

Although several pathways have been proposed, the underlying mechanisms responsible for VPA-induced hepatic steatosis remain inadequately understood. The proposed pathways include the generation of the hepatotoxic metabolite 4-ene-VPA 6, inhibition of fatty acid β-oxidation 7, induction of fatty acid absorption and triglyceride (TG) synthesis owing to the suppression of carnitine palmitoyltransferase 1 levels 8. Although L-carnitine supplementation was initially considered a potential approach to mitigating the hepatotoxic effects of VPA through its influence on fatty acid β-oxidation 9, subsequent studies have not substantiated its significant efficacy in ameliorating VPA-induced toxicity 10.

The importance of nuclear factor E2-related factor 2 (NRF2) in hepatic steatosis has garnered increasing attention in recent years. Under normal conditions, NRF2 functions as a hepatoprotective factor by regulating cellular oxidative stress (OS) responses and maintaining intracellular redox homeostasis. However, hyperactivation of NRF2 promotes lipid accumulation, as evidenced by the elevated NRF2 levels observed in clinical cases of non-alcoholic fatty liver disease 11, 12. The outcome is further supported by findings demonstrating that mice overexpressing NRF2 exhibited increased hepatic lipid accumulation, regardless of being fed a low-fat or high-fat diet 13. In contrast, *Nfe2l2* knockout (*Nfe2l2*^KO^) mice that were fed a high-fat diet demonstrated reduced liver weight and TG content compared with wild-type (WT) mice 14, 15. Notably, a similar increase in NRF2 levels was observed after VPA administration. Rats administrated high-dose VPA treatment for 15 d exhibited elevated NRF2 levels in the lung tissue, along with increased nuclear expression 16.

In this study, we aimed to comprehensively explore the impact of NRF2 on VPA, as well as the correlation between NRF2 and hepatic steatosis. Our results demonstrated that VPA induced NRF2 nuclear translocation in liver, and this translocation was positively correlated with TG levels. Further investigations revealed that VPA could bind to specific sites (Cys288 and Arg415) on Kelch-like ECH-associated protein 1 (KEAP1), triggering autophagic degradation and disrupting the KEAP1-NRF2 complex. The observed mechanism facilitated NRF2 nuclear translocation and subsequently activated the transcription of its downstream target, fatty acid transport protein 2 (FATP2), ultimately accelerating the onset of hepatic steatosis. Our findings indicate that FATP2 plays a crucial role in NRF2-mediated fatty acid metabolism. Therefore, inhibiting the NRF2-FATP2 axis may represent a promising therapeutic approach for drug-induced fatty liver disease.

## Results

### Elevated NRF2 levels were correlated with development of VPA-induced hepatic steatosis

**Figure 1:**
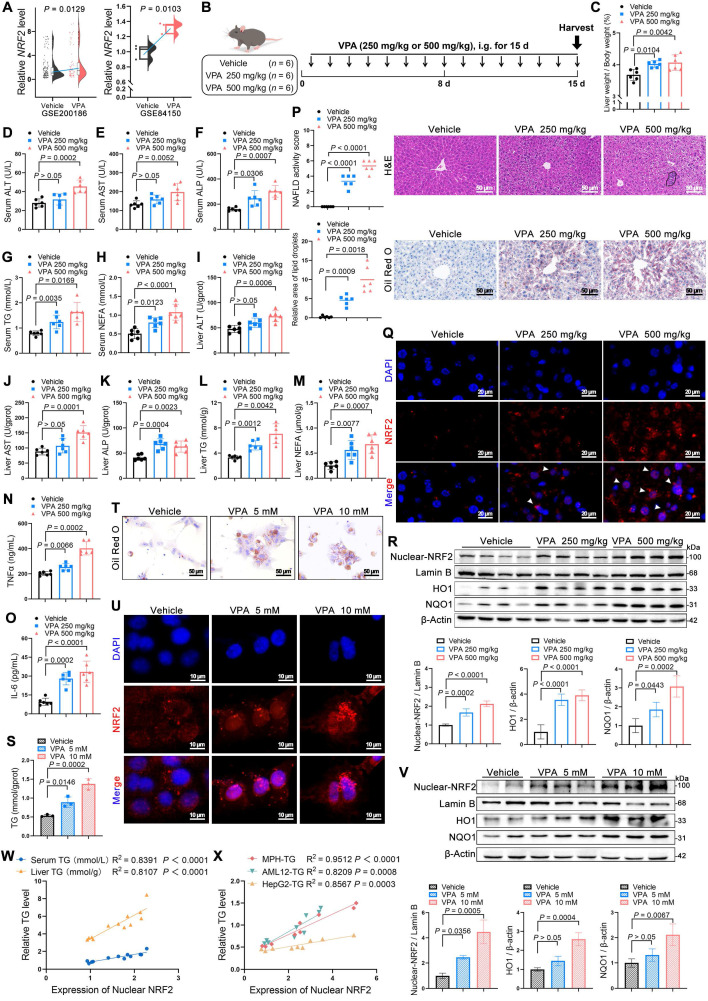
** Correlations between NRF2 levels and the development of valproic acid-induced hepatic steatosis. (A)** Relative *NRF2* levels of VPA-induced hepatic steatosis in human hepatocytes from the GEO database (GSE200186 and GSE84150). **(B)** Schedule of VPA-induced hepatic steatosis in mice. Mice in VPA groups were administered VPA-Na (250 mg/kg or 500 mg/kg body weight) through gavage daily for 15 d, whereas mice in the vehicle group were administered equal amounts of vehicle solution (PBS). **(C)** Liver coefficient (liver weight/body weight) of mice. **(D**-**H)** Serum ALT, AST, ALP, TG and NEFA level.** (I-M)** Liver ALT AST, ALP, TG and NEFA level. **(N, O)** Expression of inflammatory cytokines TNFα and IL-6 in serum. *N* = 6 mice per group in (**C-O**). **(P)** H&E and oil red O staining of liver with histopathological scoring. Scale bar, 50 μm, *n* = 6 mice per group. **(Q)** Immunofluorescence staining of NRF2 (red) expression levels in liver. Scale bar, 20 μm, *n* = 3 mice per group. **(R)** Protein expression of nuclear NRF2, HO1, and NQO1 in liver.* n* = 4 mice per group. **(S)** TG level of MPHs. **(T)** Oil red O staining of MPHs. Scale bar, 50 μm. **(U)** Immunofluorescence staining of NRF2 (red) expression levels in MPHs. Scale bar, 10 μm. **(V)** Protein expression of nuclear NRF2, HO1, and NQO1 in MPHs. *N* = 3 biologically independent samples in (**S-V**). **(W)** Correlation analysis of nuclear NRF2 expression and serum TG level in mice, nuclear NRF2 and liver TG level in mice.* n* = 4 biologically independent samples. **(X)** Correlation analysis of nuclear NRF2 expression and TG level in MPHs, nuclear NRF2 expression and TG level in AML12 cells, and nuclear NRF2 expression and TG level in HepG2 cells. *n* = 3 biologically independent samples. Statistical significance was determined using one-way analysis of variance. Data are presented as mean ± SEM.

First of all, an elevated *NRF2* expression was observed in VPA-treated hepatocytes in the Gene Expression Omnibus (GEO) database (Figure 1A). Subsequently, mice were administered two doses of VPA (250 and 500 mg/kg) to further explore the possible function of NRF2 in VPA-induced hepatic steatosis (Figure 1B). Both low- and high-dose VPA groups exhibited hepatotoxicity, evidenced by an increased liver coefficient (liver weight/body weight) compared with that of the control group (Figure 1C). In addition, serum and liver tissue levels of alanine aminotransferase (ALT), aspartate aminotransferase (AST), alkaline phosphatase (ALP), TG, and non- esterified‌ fatty acid (NEFA) were increased to varying degrees in both treatment groups (Figure 1D-M), accompanied by inflammatory responses (Figure 1N, O). Histological analysis using hematoxylin and eosin (H&E) and Oil Red O staining revealed more severe microvesicular steatosis, along with lobular inflammation and lipid deposition, in the high-dose VPA group than in the control and low-dose VPA groups (Figure 1P). Consistent with lipid deposition, NRF2 nuclear translocation in liver tissue exhibited a dose-dependent pattern (Figure 1Q, R). This VPA-induced hepatic steatosis accompanied by increased NRF2 nuclear translocation, was further confirmed *in vitro* using mouse primary hepatocytes (MPHs) (Figure 1S-V) (Figure S1A-F). Further analysis revealed a strong positive correlation between NRF2 nuclear expression and TG content (Figure 1W, X).

### NRF2 overexpression aggravated VPA-induced hepatic steatosis and tissue damage

**Figure 2:**
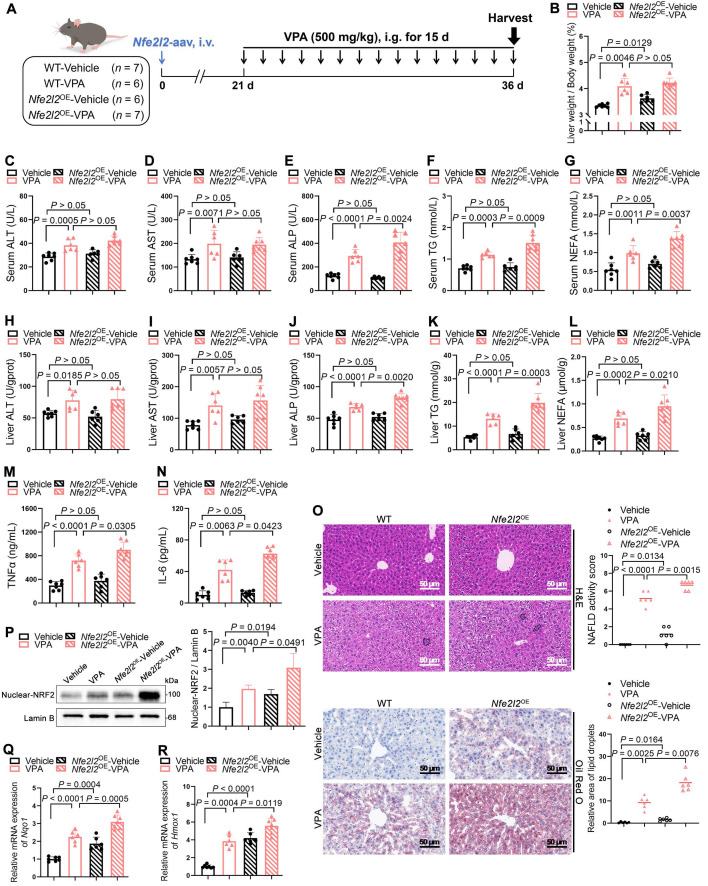
** Effects of NRF2 overexpression on VPA-induced hepatic steatosis and tissue damage. (A)** Schedule of VPA-induced hepatic steatosis in mice. Mice in *Nfe2l2*^OE^ groups were obtained by injecting 5 × 10^11^ *v*.*g*. *Nfe2l2*-aav (diluted in PBS) into the tail vein of mice 3-4 weeks before the designated experiments, whereas mice in WT group were administered equal amounts of vehicle solution. Mice in VPA groups were administered the VPA-Na (500 mg/kg body weight) through gavage daily for 15 d, whereas mice in vehicle group were administered equal amounts of vehicle solution.** (B)** Liver coefficient (liver weight/body weight) of mice. **(C**-**G)** Serum ALT, AST, ALP, TG and NEFA level.** (H**-**L)** Liver ALT AST, ALP, TG and NEFA level. **(M, N)** Expression of inflammatory cytokines TNFα and IL-6 in serum. WT-Vehicle group, *n* = 7 mice, WT-VPA group, *n* = 6 mice, *Nfe2l2*^OE^-Vehicle group, *n* = 6 mice and *Nfe2l2*^OE^-VPA group, *n* = 7 mice in **(B**-**N**). **(O)** H&E and oil red O staining of liver with histopathological scoring. Scale bar, 50 μm, *n* = 6 mice per group. **(P)** Proteins expression of nuclear NRF2. *n* = 3 mice per group.** (Q, R)** mRNA expression of *Hmox1* and *Nqo1* in liver. *n* = 6 mice per group in (**Q, R**). Statistical significance was determined using one-way analysis of variance. Data are presented as mean ± SEM.

An adeno-associated virus-expressing *Nfe2l2* (*Nfe2l2*-aav) was constructed to achieve liver-specific overexpression of NRF2 in mice to further explore the crucial role of NRF2 in VPA-induced hepatic steatosis. The qPCR analysis of liver samples from mice injected with either empty vector control or *Nfe2l2*-aav showed that *Nfe2l2*-aav significantly increased NRF2 mRNA levels by over 10-fold, with a corresponding increase in protein levels (Figure S2A-D). Subsequently, mice with successful NRF2 overexpression (*Nfe2l2*^OE^) and their littermate WT counterparts were exposed (or not exposed) to VPA (Figure 2A).

In the absence of VPA, *Nfe2l2*^OE^ -Vehicle mice exhibited an increase in liver coefficient. However, compared with WT-VPA mice, *Nfe2l2*^OE^-VPA mice exhibited no significant change in liver coefficient (Figure 2B). Despite the lack of significant changes following NRF2 overexpression alone, *Nfe2l2*^OE^ mice exposed to VPA demonstrated significant elevation in serum and liver tissue levels of ALP, TG and NEFA (Figure 2C-L), as well as in the expression of inflammatory cytokines including tumor necrosis factor-alpha (TNFα) and interleukin-6 (IL-6) (Figure 2M, N). These changes were accompanied by exacerbated pathological damage and lipid deposition in liver tissue (Figure 2O). Consistently, both VPA treatment and *Nfe2l2*-aav stimulation significantly increased nuclear NRF2 protein levels, as well as mRNA levels of its downstream genes *Hmox1* and *Nqo1* (Figure 2P-R*)*.

Additionally, primary hepatocytes isolated from mice with successful NRF2 overexpression were treated with VPA *in vitro*, which also revealed that NRF2 overexpression exacerbated VPA-induced lipid deposition (Figure S2E, F).

### NRF2 knockout attenuated VPA-induced hepatic steatosis and tissue damage

**Figure 3:**
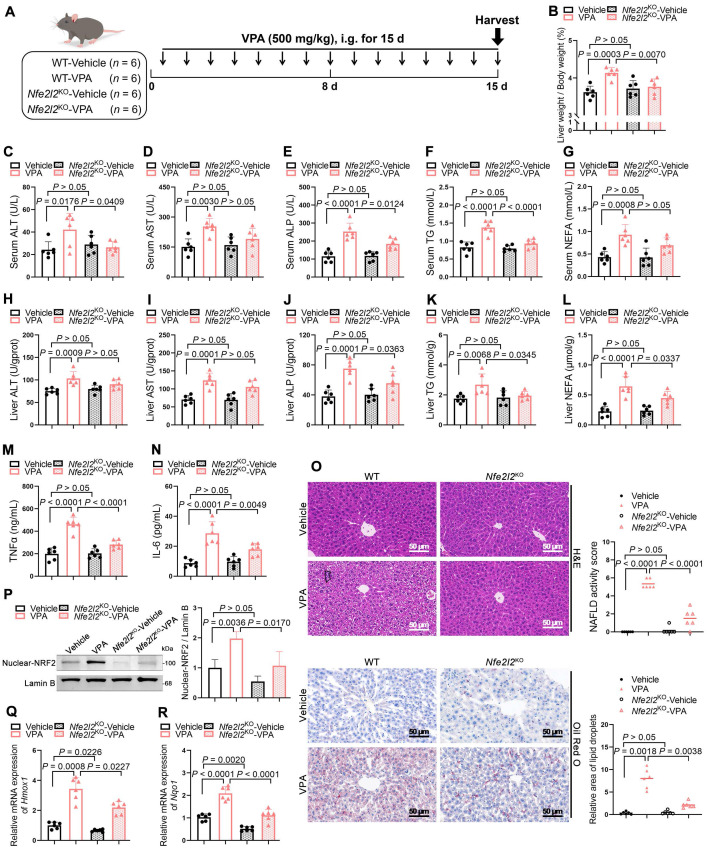
** Effects of NRF2 knockout on VPA-induced hepatic steatosis and tissue damage. (A)** Schedule of VPA-induced hepatic steatosis in mice. Mice in VPA groups were administered the VPA-Na (500 mg/kg body weight) through gavage daily for 15 d, whereas mice in vehicle group were administered equal amounts of vehicle solution.** (B)** Liver coefficient (liver weight/body weight) of mice. **(C**-**G)** Serum ALT, AST, ALP, TG and NEFA level.** (H**-**L)** Liver ALT AST, ALP, TG and NEFA level. **(M, N)** Expression of inflammatory cytokines TNFα and IL-6 in serum. *n* = 6 mice per group in (**B**-**N**). **(O)** H&E and oil red O staining of liver with histopathological scoring. Scale bar, 50 μm, *n* = 6 mice per group. **(P)** Protein expression of nuclear NRF2. *n* = 3 mice per group.** (Q, R)** mRNA expression of *Hmox1* and *Nqo1* in liver. *n* = 6 mice per group in (**Q, R**). Statistical significance was determined using one-way analysis of variance. Data are presented as mean ± SEM.

*Nfe2l2*^KO^ mice were generated to determine whether the absence of NRF2 could ameliorate VPA-induced hepatic steatosis (Figure S3A-D). Under similar conditions (Figure 3A), *Nfe2l2* knockout had minimal effect on liver coefficient of mice not exposed to VPA; however, following VPA exposure, *Nfe2l2*^KO^ mice exhibited a reduction in the VPA-induced elevated liver coefficient (Figure 3B). Similarly, compared with their littermate WT-VPA counterparts, VPA-treated* Nfe2l2*^KO^ mice showed significant decreases in serum levels of ALT, ALP, and TG, as well as in liver tissue levels of ALP, TG and NEFA (Figure 3C-L). The expression of TNFα and IL-6 was also significantly reduced (Figure 3M, N). Moreover, *Nfe2l2*^KO^ mice effectively alleviated VPA-induced microvesicular steatosis, inflammatory responses, and lipid deposition (Figure 3O). *Nfe2l2* knockout inhibited the VPA-induced upregulation of nuclear NRF2 protein and its downstream targets *Hmox1* and *Nqo1* mRNA levels (Figure 3P-R). Furthermore, transfection of MPHs with si*Nfe2l2* led to effective mitigation of VPA-induced lipid deposition (Figure S3E-H).

### NRF2 mediated FATP2 transcription to promote fatty acid uptake

**Figure 4:**
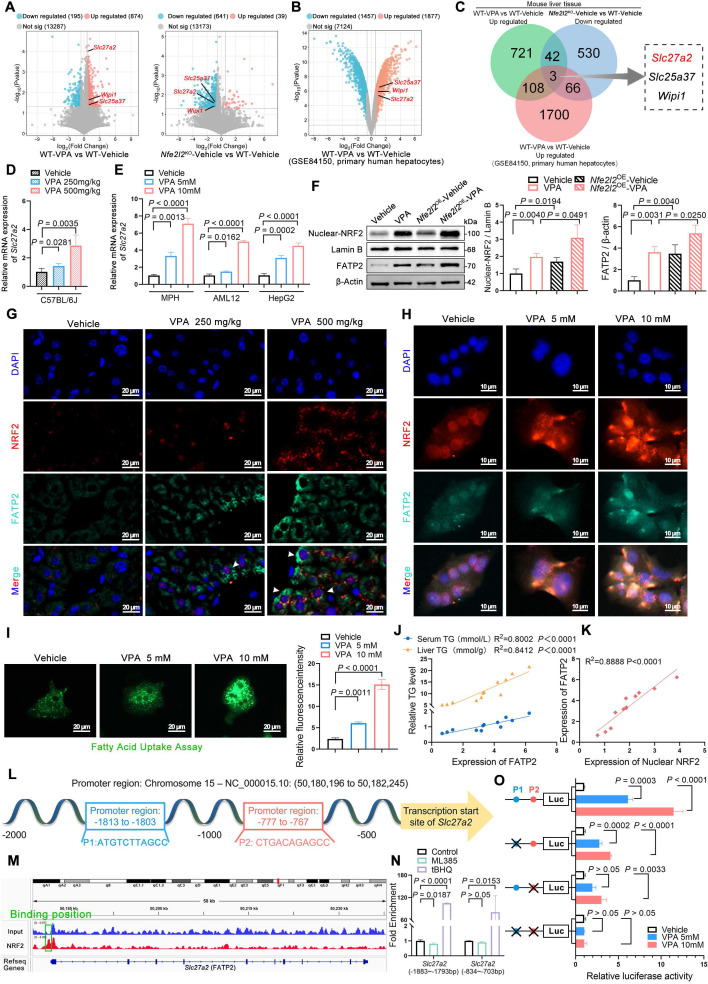
** Effects of NRF2-mediated FATP2 transcription on fatty acid uptake. (A)** RNA-seq was performed on livers of WT-Vehicle, WT-VPA, *Nfe2l2*^KO^-Vehicle and *Nfe2l2*^KO^-VPA mice (*n* = 3 per group). Volcano plot of significantly up- and downregulated genes in WT-VPA vs WT-Vehicle and *Nfe2l2*^KO^-Vehicle vs WT-Vehicle. **(B)** Volcano plot of significantly up- and downregulated genes in the GEO dataset GSE84150. **(C)** DEGs among upregulated genes from WT-VPA vs WT-Vehicle, downregulated genes from *Nfe2l2*^KO^-Vehicle vs WT-Vehicle, and upregulated genes from WT-VPA vs WT-Vehicle in the GEO dataset. **(D)** mRNA expression of *Slc27a2* in liver. *n* = 6 per group. **(E)** mRNA expression of *Fatp2* in MPHs, AML12, and HepG2 cells. *n* = 3 biologically independent samples. **(F)** Protein expression of nuclear NRF2 and FATP2 in mice. **(G)** Immunofluorescence staining of NRF2 (red) and FATP2 (turquoise) expression levels in liver. Scale bar, 20 μm. *n* = 3 mice per group in (**F, G**). **(H)** Immunofluorescence staining of NRF2 (red) and FATP2 (turquoise) expression levels in MPHs. Scale bar, 10 μm. **(I)** Fatty acid uptake assay in MPHs. Scale bar, 20 μm. *n* = 3 biologically independent samples in (**H, I**). **(J)** Correlation analysis of FATP2 expression and serum TG level in mice, FATP2 expression and liver TG level in mice. **(K)** Correlation analysis of FATP2 and nuclear NRF2 expression. **(L)** A NRF2 binding predictive analysis of the promoter region of *Slc27a2* between -2000 and +50. Two potential NRF2-antioxidant response elements were identified including P1 (-1813 to -1803): ATGTCTTAGCC and P2 (-777 to -767): CTGACAGAGCC. **(M)** Map of the *Slc27a2* locus revealing NRF2 binding in MPHs upon tBHQ treatment. The result was visualized using R. The signal of the IgG or Anti-NRF2 is represented with blue and red peaks, respectively. **(N)** Enrichment of NRF2 on the promoter region of *Slc27a2* in -1883 to -1793 and -834 to -703 upon tBHQ and ML385 treatment. **(O)** Relative luciferase activity was measured upon VPA treatment, using Renilla luciferase as a control. Comparisons were made among the WT group and FATP2 mutants harboring mutations at the P1 site, the P2 site, and both the P1 and P2 sites. Statistical significance was determined using one-way analysis of variance. Data are presented as mean ± SEM.

To systematically and comprehensively identify pivotal signaling molecules contributing to the progression of VPA-induced hepatic steatosis, mRNA microarray analysis was performed on the liver tissues of WT and *Nfe2l2*^KO^ mice. Subsequently, the transcriptome data were cross-referenced with previously published data (GSE84150 from human primary hepatocytes) to identify differentially expressed genes (DEGs). The analysis revealed that compared with that in the WT-vehicle mice, significant upregulation was observed in the expression of 874 genes in WT mice after VPA administration. However, when *Nfe2l2* was knocked out, the expression of 641 genes was significantly downregulated in the *Nfe2l2*^KO^-Vehicle group compared to that in the WT-Vehicle mice. Additionally, VPA treatment contributed to significant upregulation in the expression levels of 1,877 genes in human primary hepatocytes (Figure 4A, B). Among the DEGs, three overlapped across all three datasets, with *Slc27a2* (also known as FATP2) being the only gene associated with fatty acid metabolism (Figure 4C) (Figure S4A).

Subsequently, the expression of FATP2 was validated both *in vivo* and *in vitro*. The results indicated VPA induced a dose-dependent upregulation in both FATP2 mRNA and protein levels. *Nfe2l2* overexpression enhanced the VPA-induced upregulation of FATP2, whereas *Nfe2l2* knockout attenuated this effect (Figure 4D-H) (Figure S4B, C). The hepatic fatty acid uptake from circulation depends primarily on fatty acid transport proteins, with passive diffusion playing a minor role 17. Fatty acid uptake experiments revealed that the cellular fatty acid uptake capacity increased in a dose-dependent manner in the presence of VPA (Figure 4I). Furthermore, FATP2 expression levels showed a strong positive correlation with both TG content and nuclear NRF2 expression levels (Figure 4J, K).

Therefore, an NRF2-binding predictive analysis of the promoter region of FATP2 (*Slc27a2*) between -2000 and +50 on NRF2 was conducted. Two potential NRF2-antioxidant response elements were identified (Figure 4L): P1 (-1813 to -1803): ATGTCTTAGCC and P2 (-777 to -767): CTGACAGAGCC. Subsequently, MPHs were subjected to NRF2-specific chromatin immunoprecipitation (ChIP) followed by ChIP-qPCR. The results showed significant binding between NRF2 and the promoter region of *Slc27a2* (Figure 4M), with strong NRF2 enrichment fragments observed at both P1 and P2 after treatment with tert-butylhydroquinone (tBHQ), an NRF2 agonist. Although enrichment was inhibited after treatment with ML385, an NRF2 inhibitor, no significant difference was observed for P2 (Figure 4N). In addition, AML12 cells were subjected to dual-luciferase reporter gene assays. VPA significantly increased the transcriptional activity of NRF2 on FATP2. When either P1 or P2 was mutated, the transcriptional activity of FATP2 decreased. Additionally, when both P1 and P2 were mutated simultaneously, transcriptional activity was almost abolished (Figure 4O). These data indicate a high correlation between NRF2-mediated transcriptional activation of FATP2 and fatty acid uptake in hepatocytes.

### NRF2 exacerbated VPA-induced hepatic steatosis dependent on FATP2

**Figure 5:**
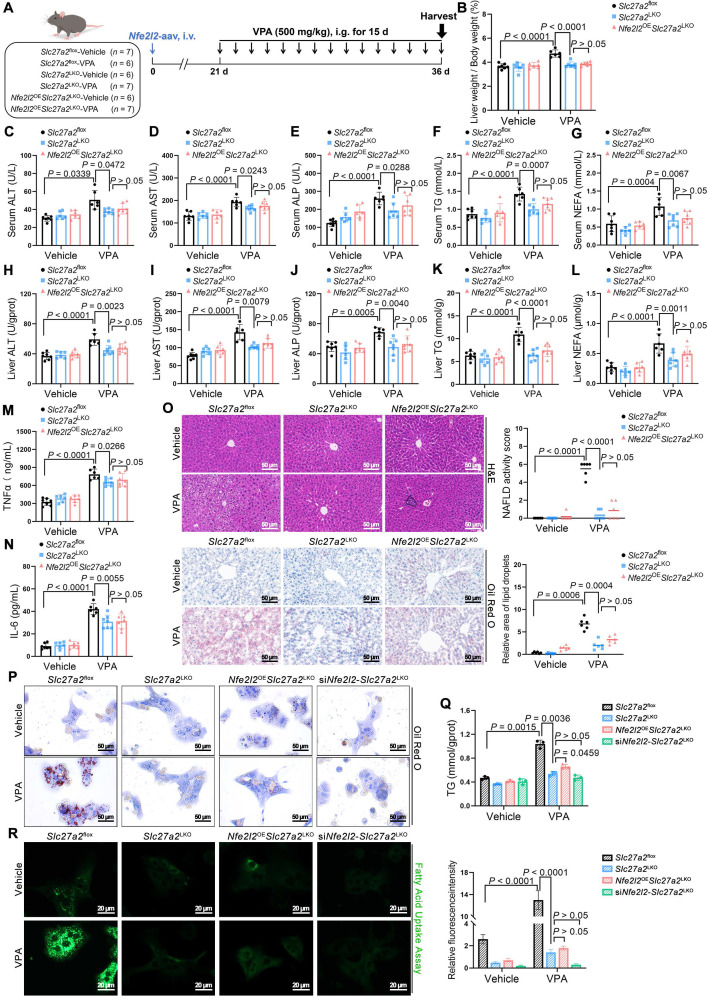
** Effects of NRF2 on VPA-induced hepatic steatosis dependent on FATP2. (A)** Schedule of VPA-induced hepatic steatosis in mice. *Slc27a2*^LKO^ mice were generated by crossing uninducible Alb-Cre mice with *Slc27a2*^flox^ mice. Mice in *Nfe2l2*^OE^* Slc27a2*^LKO^ groups were obtained by injecting 5 × 10^11^ *v*.*g*. *Nfe2l2*-aav (diluted in PBS) into the tail vein of* Slc27a2*^LKO^ mice 3-4 weeks before the designated experiments, whereas mice in* Slc27a2*^flox^ and *Slc27a2*^LKO^ groups were administered equal amounts of vehicle solution. Mice in VPA groups were administered the VPA-Na (500 mg/kg body weight) through gavage daily for 15 d, whereas mice in vehicle group were administered equal amounts of vehicle solution. **(B)** Liver coefficient (liver weight/body weight) of mice. **(C**-**G)** Serum ALT, AST, ALP, TG and NEFA level.** (H**-**L)** Liver ALT AST, ALP, TG and NEFA level. **(M, N)** Expression of inflammatory cytokines TNFα and IL-6 in serum. *Slc27a2*^flox^-Vehicle group, *n* = 7 mice, *Slc27a2*^flox^-VPA group, *n* = 6 mice, *Slc27a2*^LKO^-Vehicle group, *n* = 6 mice and *Slc27a2*^LKO^-VPA group, *n* = 7 mice,* Nfe2l2*^OE^*Slc27a2*^LKO^-Vehicle group, *n* = 6 mice and *Nfe2l2*^OE^* Slc27a2*^LKO^-VPA group, *n* = 7 mice in (**B**-**N**). **(O)** H&E and oil red O staining of liver with histopathological scoring. Scale bar, 50 μm, *n* = 6 mice per group. **(P)** Oil red O staining of MPHs. Scale bar, 50 μm. si*Nfe2l2-Slc27a2*^LKO^ group MPHs was obtained by transducing si*Nfe2l2* into primary hepatocytes extracted from *Slc27a2*^LKO^ mice. **(Q)** TG level of MPHs. **(R)** Fatty acid uptake assay in MPHs. Scale bar, 20 μm. *n* = 3 biologically independent samples in (**P**-**R**). Statistical significance was determined using one-way analysis of variance. Data are presented as mean ± SEM.

To determine the necessity of FATP2 in VPA-induced hepatic steatosis, *Slc27a2* liver-specific knockout (*Slc27a2*^LKO^) mice were generated by crossing Alb-Cre mice with *Slc27a2*^flox^ mice. Initially, gel electrophoresis was performed to confirm gene knockout efficiency (Figure S5A-C). The following day, *Nfe2l2*^OE^*Slc27a2*^LKO^ mice were injected with *Nfe2l2*-aav to achieve NRF2 overexpression (Figure 5A). Compared with their littermate control counterparts, VPA-exposed *Slc27a2*^LKO^ mice showed significantly lower liver coefficients (Figure 5B), as well as ALT, AST, ALP, TG and NEFA levels in serum and liver tissue (Figure 5C-L). Additionally, the expression of TNFα and IL-6 was significantly decreased in these mice (Figure 5M, N). Moreover, FATP2 knockout led to a reduction in microvesicular steatosis, inflammatory infiltration, and lipid deposition in liver tissue (Figure 5O). Notably, NRF2 overexpression did not reverse these effects. Further validation using MPHs indicated that both *Slc27a2*^LKO^ and si*Nfe2l2-Slc27a2*^LKO^ mice exhibited reduced VPA-induced lipid deposition and fatty acid uptake capacity (Figure 5P-R).

### VPA bound to KEAP1 leading to nuclear NRF2 entry

**Figure 6:**
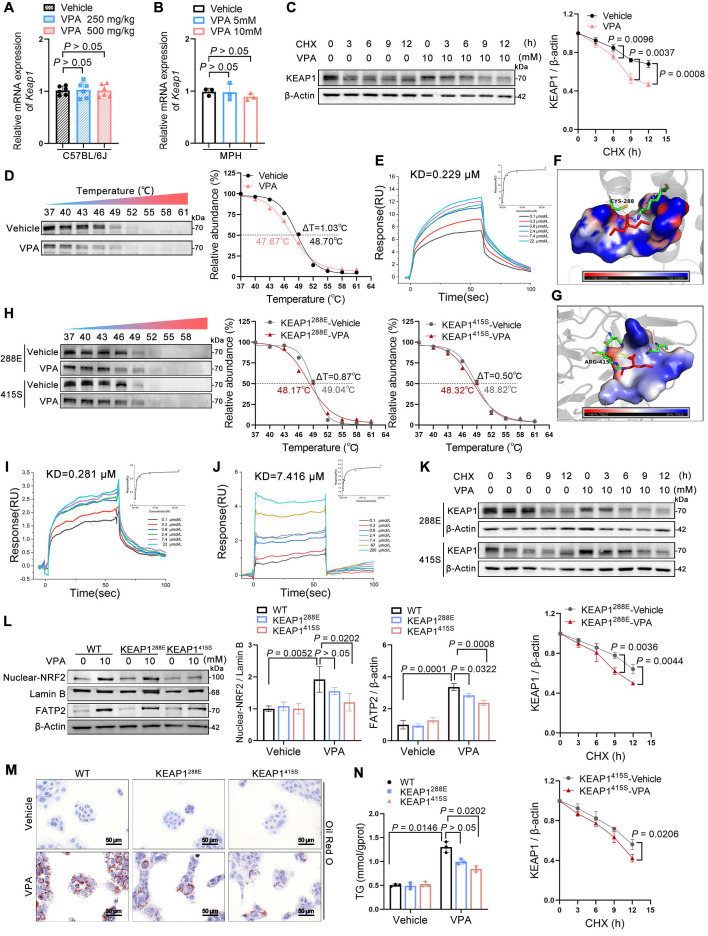
** Effects of VPA binding to KEAP1 on nuclear NRF2 entry. (A)** mRNA expression of *Keap1* in liver. *n* = 6 mice per group. **(B)** mRNA expression of *Keap1* in AML12 cells. **(C)** Protein expression of KEAP1 in AML12 cells after exposure to CHX for different durations. **(D)** Protein thermal stability after exposure (or not) to VPA in AML12 cells. *n* = 3 biologically independent samples in (**B**-**D**). **(E)** Interaction affinity between KEAP1 protein and VPA. **(F, G)** Molecular docking simulations between KEAP1 protein in CYS288 site and ARG415 site and VPA. **(H)** Protein thermal stability after exposure (or not) to VPA in AML12 cells. *n* = 3 biologically independent samples.** (I, J)** Interaction affinity between KEAP1 (CYS288 was mutated to glutamate or ARG415 was mutated to serine) protein and VPA. **(K)** Protein expression of KEAP1 after mutation in AML12 cells after exposure to CHX for different durations. **(L)** Protein expression of nuclear NRF2 and FATP2 in AML12 after mutation of specific KEAP1 sites. *n* = 3 biologically independent samples. **(M)** Oil red O staining of AML12 cells-KEAP1 mutation. Scale bar, 50 μm. **(N)** TG level of AM12 cells-KEAP1 mutation. *n* = 3 biologically independent samples in (**K**-**N**). Statistical significance was determined using one-way analysis of variance. Data are presented as mean ± SEM.

The following investigation focused on the mechanism through which VPA regulates NRF2 expression. Under normal states, NRF2 is bound and inhibited by KEAP1, which mediates its ubiquitination and subsequent degradation via the 26S proteasome; therefore, KEAP1 levels determine the stability of NRF2 18. qPCR analysis showed that the influence of VPA on the mRNA levels of KEAP1 *in vivo* and *in vitro* was insignificant, even across various concentration gradients, suggesting that VPA does not downregulate KEAP1 expression through transcription (Figure 6A, B). To determine if VPA-induced degradation of KEAP1 is a post-translational regulatory event, AML12 cells were subjected to cycloheximide (CHX), a protein translation inhibitor. After being exposed to CHX, the half-life of KEAP1 in VPA-treated cells was shorter than that in untreated cells, indicating that VPA-triggered downregulation of KEAP1 expression was primarily controlled at the protein level (Figure 6C).

Notably, VPA could bind directly to KEAP1 (Figure 6D, E) with considerable affinity, exhibiting a KD value of approximately 0.229 μM. An attempt was made to obtain co-crystals of VPA and KEAP1 to investigate their binding mode; however, this effort was unsuccessful. Subsequently, molecular docking simulations using the AutoDock software indicated that VPA could stably bind to either Cys288 or Arg415 on KEAP1 (Figure 6F, G) (Table S1). Therefore, Cys288 and Arg415 were mutated individually. As expected, this process led to a significant reduction in VPA binding to KEAP1 (Figure 6H). When Cys288 was mutated to glutamate, its KD value was 1.23 times that of the WT. However, when Arg415 was mutated to serine, its KD value was 32.38 times that of the WT (Figure 6I, J). Next, the effect of the mutations in Cys288 and Arg415 on the expression of KEAP1 was assessed. In the presence of CHX, the Arg415 mutation significantly slowed the half-life of KEAP1 following VPA treatment, whereas the change observed with Cys288 mutation was relatively modest (Figure 6K). Further analysis revealed that mutations in Cys288 and Arg415 inhibited VPA-induced NRF2 nuclear translocation and FATP2 expression to varying degrees (Figure 6L) and significantly improved hepatic steatosis (Figure 6M, N).

### VPA enhanced NRF2 expression via promotion of KEAP1 autophagic degradation

**Figure 7:**
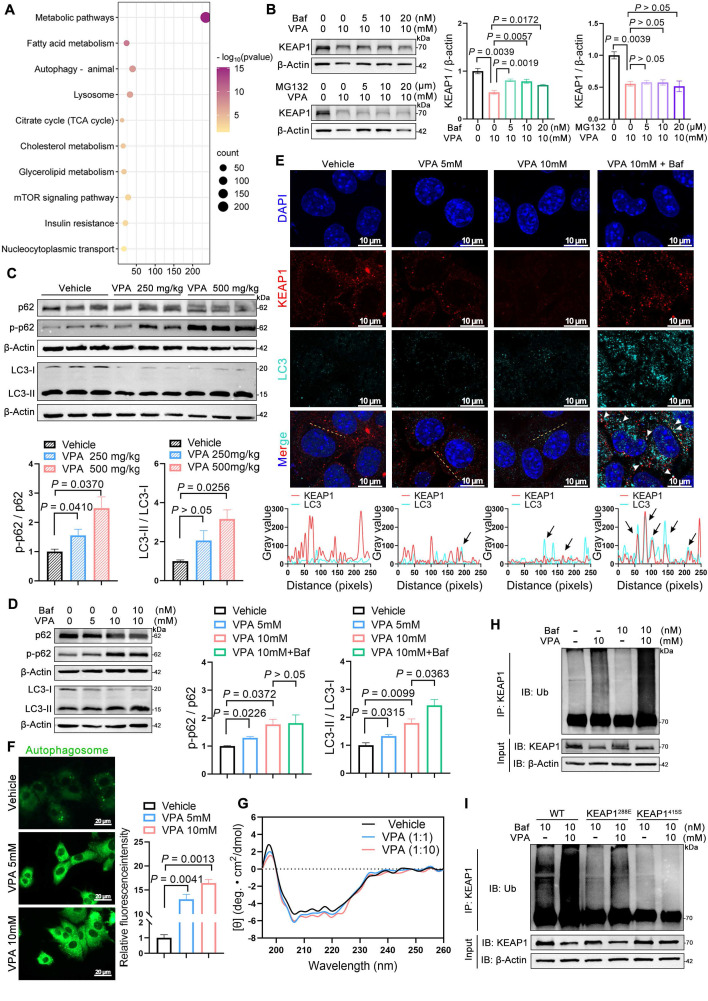
** VPA enhances NRF2 expression via promoting KEAP1 autophagic degradation. (A)** KEGG enrichment analysis of differentially expressed genes in the WT-Vehicle *vs.* WT-VPA groups.** (B)** Protein expression of KEAP1 upon Baf or MG132 treatment in AML12 cells. *n* = 3 biologically independent samples in **(B)**.** (C)** Protein expression of p62, p-p62, and LC3 in liver. *n* = 3 mice per group in **(C)**.** (D)** Protein expression of p62, p-p62, and LC3 in AML12 cells. **(E)** Immunofluorescence staining of KEAP1 (red) and LC3 (turquoise) expression levels with co-localization analysis in AML12 cells. Scale bar, 10 μm. **(F)** Dansylcadaverine staining in AML12 cells. Scale bar, 20 μm. **(G)** Circular dichroism spectroscopic analysis of VPA-KEAP1 binding interactions. Vehicle, treated control; VPA (1:1), KEAP1:VPA = 1:1 molar ratio; VPA (1:10), KEAP1:VPA = 1:10 molar ratio. **(H, I)** Ubiquitination assay of KEAP1 in AML12 cells. *n* = 3 biologically independent samples in **(D-I)**. Statistical significance was determined using one-way analysis of variance. Data are presented as mean ± SEM.

KEGG pathway analysis (Figure 7A) of hepatic transcriptomes from WT-vehicle vs. WT-VPA groups revealed significant enrichment in autophagy-related pathways (autophagy-animal, lysosome, and mTOR signaling), prompting focused examination of autophagic regulation. To elucidate the pathway involved in VPA-mediated KEAP1 degradation, AML12 cells were treated with the autophagosome inhibitor bafilomycin A1 (Baf) or the proteasome inhibitor MG132 in combination with VPA. VPA-induced degradation of KEAP1 in AML12 cells was rescued by Baf but not by MG132 (Figure 7B). The finding indicates that VPA promoted KEAP1 degradation through an autophagic mechanism, which could be inhibited by an autophagosome inhibitor; however, the degradation was not affected by the proteasome inhibitor.

p62 contains both an LC3-interacting region and a KEAP1-interacting region (KIR), enabling its dual binding to LC3 on autophagosomal membranes and cytoplasmic KEAP1 19, 20. Phosphorylation of the KIR domain enhances p62's affinity for KEAP1, leading to autophagic degradation of the p62-KEAP1 complex. This process stabilizes NRF2, facilitating its nuclear translocation to activate downstream targets 21, 22. Immunoblotting demonstrated VPA-induced p62 phosphorylation (p-p62) and elevated LC3-II/LC3-I ratios (Figure 7C, D), confirming autophagic activation. Co-localization of KEAP1 with LC3 was enhanced by Baf pretreatment (Figure 7E), whereas dansylcadaverine staining revealed increased autophagosome formation under VPA exposure (Figure 7F).

Cellular stress induces the accumulation of misfolded proteins that undergo ubiquitination and subsequent recognition by p62, which serves as a selective autophagy receptor. This ubiquitin-tagged cargo is then chaperoned to autophagosomes through p62-LC3 interactions, with final degradation achieved via autophagosome-lysosome fusion 23. However, circular dichroism spectroscopy excluded significant VPA-induced structural alterations in KEAP1 (Figure 7G). Surface plasmon resonance and cellular thermal shift assays confirmed VPA binding to KEAP1's Cys288 and Arg415 residue (Figure 6D-J). We postulate this interaction exposes latent ubiquitination sites (e.g., Lys254/287 near Cys288, and Lys333/615 near Arg415 masked by NRF2), enabling ubiquitin-mediated autophagic targeting.

Consistent with the model above, VPA treatment increased KEAP1 ubiquitination, which was amplified by Baf-induced autophagic blockade (Figure 7H). Cys288 and Arg415 mutation attenuated VPA-driven KEAP1 ubiquitination (Figure 7I), confirming the structural dependency. The findings establish the following mechanism: 1) VPA binding induces conformational exposure of KEAP1 ubiquitination sites; 2) Ubiquitinated KEAP1 recruits phosphorylated p62; 3) p62-KEAP1-LC3 interactions mediate autophagic degradation, enabling NRF2 stabilization and transcriptional activation of lipogenic targets.

### Inhibition of NRF2 expression ameliorates VPA-induced hepatic steatosis

**Figure 8:**
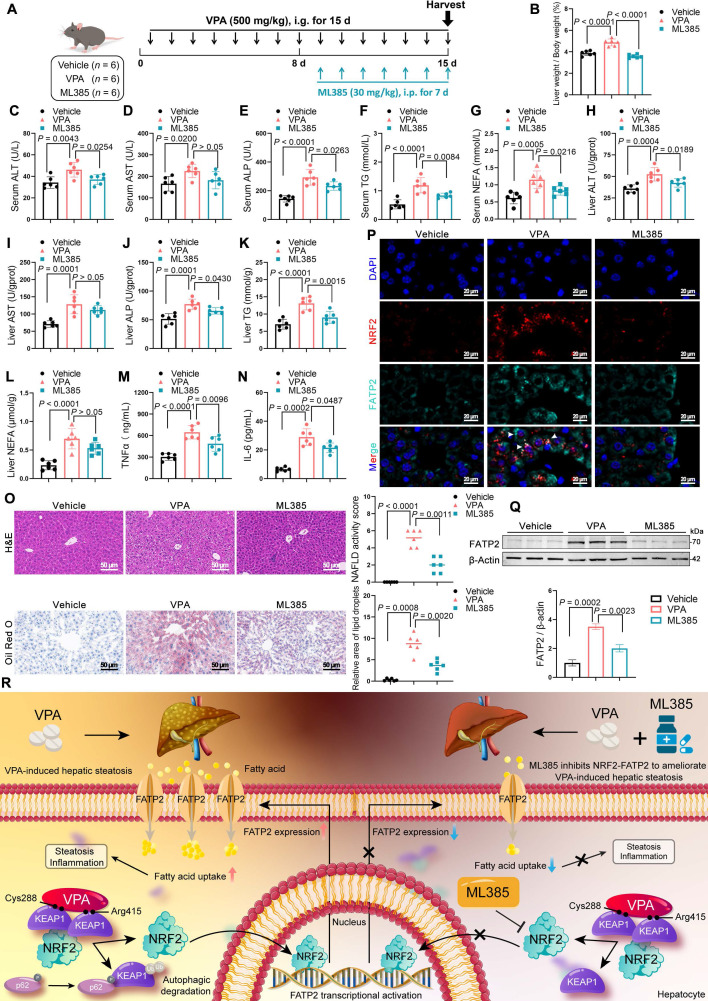
** Effects of inhibition of NRF2 expression on VPA-induced hepatic steatosis. (A)** Schedule of VPA-induced hepatic steatosis in mice. Mice in VPA and ML385 groups were administered the VPA-Na (500 mg/kg body weight) through gavage daily for 15 d. Mice in ML385 group were administered the ML385 (30 mg/kg body weight) through gavage daily for the last 7 d. Mice in vehicle group were administered equal amounts of vehicle solution. **(B)** Liver coefficient (liver weight/body weight) of mice. **(C**-**G)** Serum ALT, AST, ALP, TG and NEFA level.** (H**-**L)** Liver ALT AST, ALP, TG and NEFA level. **(M, N)** Expression of inflammatory cytokines TNFα and IL-6 in serum. **(O)** H&E and oil red O staining of liver with histopathological scoring. Scale bar, 50 μm. *n* = 6 mice per group in (**B**-**O**). **(P)** Immunofluorescence staining of NRF2 (red) and FATP2 (turquoise) expression levels in liver. Scale bar, 20 μm. **(Q)** Protein expression of FATP2. *n* = 3 mice per group in **(P, Q)**. **(R)** A schematic illustrating how ML385 inhibits NRF2-FATP2 to ameliorate VPA-induced hepatic steatosis. Statistical significance was determined using one-way analysis of variance. Data are presented as mean ± SEM.

ML385 is a specific inhibitor of NRF2 that inhibits NRF2-mediated transcriptional activity by selectively binding to the Neh1 domain 24. Therefore, the therapeutic potential of ML385 in mice with VPA-induced hepatic steatosis was evaluated (Figure 8A). ML385 reduced the abnormally elevated liver coefficient (Figure 8B) and decreased the expression of ALT, ALP, TG in both serum and liver tissue, although its effect on AST was not significant (Figure 8C-L). In addition, ML385 mitigated inflammatory responses (Figure 8M, N), improved pathological damage, and decreased lipid deposition in the liver (Figure 8O). Immunofluorescence staining and western blot results further indicated that the presence of ML385 led to a downregulation of both NRF2 and FATP2. (Figure 8P, Q). These results suggest that the NRF2 inhibitor ML385 can reduce NRF2 and FATP2 levels, thereby resulting in a reduction in fatty acid absorption and an amelioration of VPA-mediated hepatic steatosis (Figure 8R).

## Discussion

VPA is extensively used to treat psychiatric disorders, and recent studies have highlighted its potential anticancer 25, 26 and autoimmune disease-improving effects 27. Hepatotoxicity stands as one of the most frequent adverse reactions associated with long-term VPA treatment 28; however, its underlying pathogenic mechanisms remain incompletely elucidated. In this study, a comprehensive evaluation was conducted to ascertain the importance of NRF2 in both the onset and development of VPA-mediated hepatic steatosis. The pathogenic mechanism was elucidated by demonstrating, for the first time, that VPA binds to KEAP1, causing NRF2 to migrate to the nucleus and subsequently activate the transcription of FATP2, ultimately increasing fatty acid uptake, which triggers hepatic steatosis.

As the master regulator of redox homeostasis, NRF2 orchestrates cytoprotective responses under physiological or acute injury conditions by binding to antioxidant response elements to upregulate detoxifying enzymes such as *Hmox1* and *Nqo1*, thereby effectively scavenging reactive oxygen species (ROS) and mitigating toxic liver injury 29-31. However, sustained NRF2 activation drives hepatic pathology through lipogenic promotion, suppressed lipolysis, pro-tumorigenic p62 interactions, and cancer-associated metabolic reprogramming 19, 32-35. The Yamamoto research group explains this dichotomy: transient and moderate NRF2 activation or partial KEAP1 knockdown confer cytoprotection 36, 37, whereas NRF2 hyperactivation, caused by constitutive overexpression, KEAP1 knockout, or gain-of-function mutations, causes metabolic imbalance and carcinogenesis 35, 37.

In the present study, continuous VPA administration led to progressive accumulation of the lipid peroxidation product malondialdehyde (MDA) and a decrease in the expression of the antioxidant enzyme superoxide dismutase (SOD), resulting in OS damage. Although NRF2 overexpression partially restored SOD and reduced MDA (Figure S6A, B), it failed to alleviate liver damage. Paradoxically, sustained NRF2 elevation amplified VPA-induced lipid accumulation, inflammation, and histopathological injury (Figure 2). Mechanistically, whereas NRF2 moderately counteracted OS, its lipid-promoting effects dominated under chronic activation. The conclusion was further validated by attenuated steatosis in NRF2-knockout models (Figure 3).

A combined analysis using transcriptomics and the GEO database was conducted to investigate how NRF2 regulates fatty acid metabolism in VPA-induced hepatic steatosis. By intersecting three datasets, three significant DEGs were identified: *Slc27a2* (FATP2), *Slc25a37*, and *Wipi1*. Among these, *Slc25a37* primarily facilitates iron transport 38, 39, whereas *Wipi1* is involved in autophagy 40. Notably, *Slc27a2* was the only gene related to fatty acid metabolism. FATP2, a membrane protein highly expressed in liver tissue, primarily facilitates the import of long-chain fatty acid 41. Studies have demonstrated that elevated FATP2 expression in polymorphonuclear myeloid-derived suppressor cells leads to higher lipid accumulation in various patients with cancer than in healthy individuals, and that its inhibitors can reduce lipid entry into these cells indicating a role in immune regulation and potential antitumor effects 42. Furthermore, FATP2 knockout has been demonstrated to improve high-fat diet-induced hepatic steatosis, leading to diminished fat accumulation in tissue culture and over 60% decrease in lipid absorption *in vivo* 43. These findings suggest that FATP2 promotes lipid uptake, thereby exacerbating the accumulation of hepatic lipids. Both *in vitro* and *in vivo* experiments confirmed these findings: VPA dose-dependently increased FATP2 expression (Figure 4D-H) (Figure S4B, C) and promoted fatty acid uptake (Figure 4I). When FATP2 was knocked out, the cellular fatty acid uptake capacity was reduced, and even NRF2 overexpression, this effect could not be reversed (Figure 5P-R).

This study had several limitations. Under normal states, NRF2 is anchored within the cytoplasm through its inactivator KEAP1. Meanwhile, acting as a vital substrate for the E3 ubiquitin ligase complex, KEAP1 promotes NRF2 ubiquitination and subsequent degradation by the proteasome. Multiple regulatory mechanisms can enhance NRF2 expression through both KEAP1-dependent and -independent pathways in response to OS or other stimuli 44. However, this study explored only the KEAP1-dependent activation pathway of NRF2. Our results revealed that VPA could bind to Cys288 located on the intervening region and Arg415 located on the Kelch region of KEAP1, disrupting the conformation of the KEAP1-NRF2 complex. Consequently, NRF2 gathered in the cytoplasm and translocated to the nucleus, driving the transcription of relevant target genes. However, NRF2 expression can also be activated through protein-protein interactions and epigenetic modifications. For instance, p21 binds directly to NRF2 and inhibits its degradation 45, and the histone demethylase PHD finger protein 2 enhances carbohydrate response element binding protein-driven NRF2 expression by promoting the demethylation of histone H3 lysine 9 dimethylation 46. Therefore, further investigation is required to determine whether VPA can regulate NRF2 expression through KEAP1-independent pathways.

In summary, our study elucidates a mechanism underlying VPA-induced hepatic steatosis. Specifically, VPA was observed to bind to the Cys288 and Arg415 residues of KEAP1, resulting in disruption to the complex of KEAP1-NRF2 and facilitating NRF2 nuclear translocation. This process triggered the transcription of the downstream target FATP2, subsequently enhancing hepatic fatty acid uptake and causing hepatic steatosis. Therefore, inhibiting the NRF2-FATP2 axis may be a potential strategy for alleviating VPA-induced hepatic steatosis (Figure 8R). Our findings offer promising perspectives regarding the regulation of fatty acid metabolism in the context of fatty liver disease induced by drugs.

## Materials and methods

### Animal treatment

Six- to eight-week-old male C57BL6/J mice were purchased from Vital River Laboratory Animal Technology Co., Ltd (Guangdong, China). *Nfe2l2*-aav and all plasmids were constructed by Tsingke Biotech Co., Ltd (Guangdong, China). The recombinant plasmid pAAV-TBG-Nfe2l2-3×FLAG-P2A-GdGreen-WPRE was constructed and co-transfected into 293T cells with AAV8 serotype packaging plasmids and pHelper plasmid to generate *Nfe2l2*-aav viral particles. *Nfe2l2*^OE^ mice were obtained by injecting 5 × 10^11^ *v*.*g*. *Nfe2l2*-aav diluted in phosphate-buffered saline (PBS, 100 µL) into the tail vein of mice 3-4 weeks before the designated experiments. *Nfe2l2*^KO^ mice were obtained from RIKEN BioResource Research Center. These mice were generated by introducing a targeting vector containing a lacZ-neo cassette into E14 ES cells, which replaced a 1.2 kb segment encompassing the remainder of exon 5 coding sequences in the *Nfe2l2* gene. *Slc27a2*^LKO^ mice were generated by crossing uninducible Alb-Cre mice with *Slc27a2*^flox^ mice.* Slc27a2*^flox^ mice were generated by Cyagen Co., Ltd (Suzhou, China) using the CRISPR/Cas9 system. All genotyping data are available in the Supplementary Information.

All mice were housed and fed at the Guangzhou University of Chinese Medicine's Experimental Animal Center under controlled environmental conditions (40-70% humidity, 22±2 °C, and 12-h light-dark alternation), and they had unrestricted access to food and water. Ethical approval for the experiments was granted by the Ethics Review Board of the School of Pharmaceutical Sciences, Guangzhou University of Chinese Medicine.

### Separation of mouse primary hepatocytes

MPHs were separated using a two-step perfusion method. In brief, mice were anesthetized using isoflurane, and their chest and abdomen were disinfected using 75% alcohol, after which they were fixed on a workbench. Subsequently, the hepatic portal vein and inferior vena cava were exposed for manipulation through surgical incision. The inferior vena cava was cannulated for perfusion with Buffer I (1% (v/v) penicillin-streptomycin [P/S] solution, 25 mM 4-(2-hydroxyethyl)-1-piperazineethanesulfonic acid [HEPES], 0.5 mM ethylene glycol tetraacetic acid, and D-Hank's solution replenished to 30 mL) at a 5 mL/min flow rate, whereas the hepatic portal vein was cut off until no blood was expelled from the liver. Afterwards, perfusion with Buffer II (1% P/S solution, 3 mM CaCl_2_, 15 mM HEPES, 100 CUD/mL type Ⅳ collagenase, and Dulbecco's modified Eagle's medium [DMEM] replenished to 30 mL) was initiated until the liver softened and lost its elasticity. The liver was then excised and placed in a dish containing cold DMEM, and non-target tissues were removed carefully. Next, the liver was filtered through a 70 μm sieve, and the filtrate was centrifuged at 50 × g for 2 min. After discarding the supernatant, the cells were then resuspended in DMEM (this step was repeated three times). In the final centrifugation, the cells were washed with 40% Percoll solution. After cell counting, the MPHs were plated, and the medium was changed after 6 h, to prepare the cells for experimentation.

### Cell culture

MPHs were separated according to the methodology described in the preceding section and cultured in DMEM containing 1% P/S solution and 10% fetal bovine serum (FBS). AML12 and HepG2 cells lines were obtained from the Cell Bank of the Chinese Academy of Sciences. AML12 cells were cultured in a medium composed of DMEM/F12 (1:1), supplemented with 1% P/S solution, 10% FBS, 40 ng/mL dexamethasone, and 1% insulin-transferrin-sodium selenite. HepG2 cells were cultured in a medium composed of DMEM, supplemented with 1% P/S solution and10% FBS. All cells were kept in a 37 °C incubator with 5% CO_2_ under humidified conditions.

### Liver function, inflammatory factor and oxidative stress level

Serum and hepatic levels of ALT, AST, ALP, and TG, as well as hepatic levels of MDA and SOD, were quantified using biochemical assay kits according to the manufacturer's instructions (Nanjing Jianchen, China). Serum levels of inflammatory factors TNFα and IL-6 were determined using ELISA kit (mlbio, China).

### Pathological staining

Mouse livers were harvested and immersed in a 4% paraformaldehyde solution for fixation. Portions of the tissue underwent standard paraffin embedding procedures, followed by H&E staining. Other liver samples were embedded in optimal cutting temperature compound, subjected to freezing, sliced into sections, and visualized using Oil Red O stain to assess lipid deposition.

MPH, AML12, and HepG2 cells seeded into 12-well plates and were fixed in 4% paraformaldehyde solution for subsequent staining with Oil Red O.

### Nonalcoholic fatty liver disease activity score

Histopathological evaluation of hepatic sections was performed using the NAFLD Activity Score system 47. This validated scoring metric quantifies disease severity through three key histopathological features:

Steatosis Grading (0-3):

0: < 5% hepatocytes affected; 1: 5-33% involvement; 2: 34-66% involvement; 3: > 66% involvement.

Lobular Inflammation Scoring (0-3):

0: No inflammatory foci per 200× magnification field; 1: < 2 foci; 2: 2-4 foci; 3: >4 foci.

Hepatocellular Ballooning Assessment (0-2):

0: Absent; 1: Few ballooned cells; 2: Numerous ballooned cells.

Per diagnostic consensus, a total NAS ≥ 5 is confirmatory for NASH, while scores ≤ 2 generally exclude NASH diagnosis.

### Real-time quantitative PCR

An RNA extraction kit (Beyotime, China) was used to isolate total RNA from both cultured cells and liver. Subsequently, a reverse transcription kit (Genesan, China) was employed to reverse-transcribe the extracted RNA into cDNA. SYBR Green (Genesan, China) was used to quantify mRNA expression and qTOWER^3^ system (Jena, Germany) was used for analysis. The internal control for normalization was glyceraldehyde-3-phosphate dehydrogenase. The 2^-ΔΔCt^ method was employed to calculate the relative mRNA expression levels. The sequences of all PCR primers used are provided in the Supplementary Materials (Table S2).

### Western blot

Cultured cells and liver tissues were lysed using a lysis buffer incorporating serine proteinase and protease inhibitors. The extracted protein samples were electrophoresed using sodium dodecyl sulfate-polyacrylamide gel electrophoresis and transferred onto polyvinylidene difluoride membranes in turn. Next, the blots were incubated with specific primary antibodies after blocking and then incubated with corresponding secondary antibodies. Blots were imaged employing a chemiluminescent horseradish peroxidase substrate and visualized using the Tanon5200CE system (Tanon, China). The antibodies used are listed in the Supplementary Materials (Table S3).

### Immunofluorescent staining

Immunofluorescence staining was conducted on paraffin-embedded liver sections. Antigen retrieval was employed using sodium citrate buffer following dewaxing and rehydrating the liver tissue sections. Next, the sections were incubated with specific primary antibodies and then incubated with corresponding fluorescently labeled secondary antibodies after bovine serum albumin blocking. An antifade mounting medium incorporating 4′,6-diamidino-2-phenylindole (DAPI) was employed to mount the slides. The cell samples were fixed with 4% paraformaldehyde, blocked, similarly incubated with specific primary antibodies and corresponding fluorescently labeled secondary antibodies, and mounted with DAPI. A fluorescence microscope was employed to visualize the images. The Supplementary Materials provides a list of the antibodies used.

### RNA-seq

Total RNA was extracted from liver tissue samples (three samples per group) and subjected to quality control. Following the enrichment of mRNA using Oligo dT, the RNA was randomly fragmented into 300 bp segments. These fragments were used as templates for converting into double-stranded cDNA through reverse transcription, followed by a series of steps including end-repair, A-tail addition, and adapter ligation. The resulting cDNA products were purified, size-sorted, and amplified using PCR to generate the sequencing library. Sequencing was performed using the NovaSeq X Plus platform. An analysis of intergroup differential gene expression was performed with the aid of DESeq2, and the screening threshold was set at |log_2_FC| ≥ 0.585 and *P* < 0.05.

### Chromatin immunoprecipitation assay

The ChIP experiment was conducted following our previously established protocol 48. After treatment of MPHs with NRF2 agonist tBHQ for 24 h, cross-linking was performed as per the standard procedure. The cells were subsequently collected, lysed, and subjected to sonication. Immunoprecipitation was performed using IgG and FATP2 antibodies, and DNA fragments were recovered during the final elution step. The resulting products were dissolved in double-distilled water for PCR and qPCR analyses.

### Fatty acid uptake assay

MPHs seeded into 24-well plates were treated with VPA for 24 h. Subsequently, the supernatant was removed, and the Fatty Acid Uptake Probe working solution (Dojindo, Japan) was added for a 15-min incubation. Next, an equal volume of Quenching Buffer (Dojindo, Japan) was added to the working solution, and the samples were analyzed using fluorescence microscopy.

### Surface plasmon resonance

Surface plasmon resonance experiments for KEAP1 were performed using Biacore T200 (Cytiva, Sweden) at 25 °C with a 30 μL/min flow rate. Immobilization of KEAP1 proteins onto CM5 chips were achieve employed amine coupling with a response unit range of 6500-7500. Ligands were passed over the chip in PBS with Tween buffer (pH 7.2-7.4), and VPA was diluted to specified concentrations in the running buffer, with a 60 s contact time and 180 s dissociation time. The data was analyzed with Biacore T200 *v*.3.0 based on a 1:1 binding model at steady-state affinity.

### Molecular docking

Given the unavailability of the complete protein crystal structure of the receptor KEAP1, the full-length sequence structure of KEAP1 was predicted using Alphafold3 (https://alphafoldserver.com). The Protein Data Bank format files for VPA were obtained from PubChem (https://pubchem.ncbi.nlm.nih.gov/). Molecular docking was executed using AutoDock *v*.4.2.6. The receptor-ligand binding significance was assessed based on the calculation of their binding energies and sites.

### Dual-luciferase reporter gene assay

Using the method described previously 49, the *Nfe2l2*-*Slc27a2* plasmid was transfected into AML12 cells for 16h. Following the removal of the medium, the cells were treated with VPA for 24 h. Adhering to the manufacturer's prescribed directions, the dual-luciferase reporter assay system (Promega, USA) was employed to measure the relative luciferase activity using Renilla luciferase as a control.

### Circular dichroism spectroscopy

Lyophilized KEAP1 protein (>90% purity) and VPA were dissolved in PBS at molar ratios of 1:1 and 1:10 (KEAP1:VPA). The mixtures were incubated at 25°C for 1 h to form KEAP1-VPA complexes. Vehicle group was contained equivalent PBS volumes without VPA. Circular dichroism measurements were performed using a Jasco J-1500 spectropolarimeter equipped with a 1 mm quartz cuvette under a nitrogen atmosphere. Spectra were recorded from 190 to 260 nm with the following parameters: 1 nm bandwidth, 50 nm/min scan speed, and three accumulations per sample. Buffer baselines were automatically subtracted from all measurements.

### Statistical analysis

To assess the normality and homogeneity of variance in the data, Kolmogorov-Smirnov and Bartlett's tests were performed, respectively. Subsequently, a one-way analysis of variance was carried out when datasets that met these criteria, followed by inter-group comparisons using Tukey's post-hoc test. Dunnett's T3 test was utilized for analysis in cases where the data were not normally distributed or exhibited unequal variance. The data were described as mean ± standard error of mean (SEM), with a significance threshold setting at *P* < 0.05. The data results were plotted using GraphPad Prism *v*.9.0.

## Supplementary Material

Supplementary figures and tables.

## Abbreviations

ALP: Alkaline phosphatase
ALT: Alanine aminotransferase
AST: Aspartate aminotransferase
ChIP: Chromatin immunoprecipitation
ELISA: Enzyme linked immunosorbent assay
FATP2: Fatty acid transporter protein 2
H&E: Hematoxylin-eosin staining
*Hmox1*: Heme oxygenase-1
IL-6: Interleukin-6
KEAP1: Kelch-like ECH-associated protein 1
KIR: KEAP1-interacting region
MDA: Malondialdehyde
MPH: Mouse primary hepatocytes
*Nqo1*: NAD(P)H:quinone oxidoreductase l
NEFA: non-esterified‌ fatty acid
*Nfe2l2*: Nuclear factor E2-related factor 2
*Nfe2l2* ^KO^: *Nfe2l2* knockout
*Nfe2l2* ^OE^: *Nfe2l2* overexpression
OS: Oxidative stress
qPCR: Quantitative Real-time PCR
ROS: Reactive oxygen species
SEM: standard error of mean
SOD: Superoxide dismutase
SPR: Surface Plasmon Resonance
tBHQ: tert-Butylhydroquinone
TG: Triglyceride
TNFα: Tumor necrosis factor α
VPA: Valproic acid
