# Supplementary material for: Mechanism of valproic acid-induced hepatic steatosis via enhancing NRF2-FATP2-mediated fatty acid uptake
Source: Theranostics. 2025 Apr 13;15(11):5258–76. doi: 10.7150/thno.108593 (PMC12036889; doi:10.7150/thno.108593)
Supplement: Supplementary file 1 — Supplementary figures and tables. [file thnov15p5258s1.pdf]

Supplementary Materials for

Mechanism of valproic acid-induced hepatic steatosis via enhancing NRF2-FATP2-mediated fatty acid uptake

Xiaoliang He[1, 2, 3], Rui Yuan[1, 2, 3], Ying Chen[1, 2, 3], Wenni Huang[1, 2], Zihao Xu[1, 2, 3], Bixia Wang[1], Changhui Liu[1, 2*], Tianqin Xiong[1, 2, 3*]

[1]School of Pharmaceutical Sciences, Guangzhou University of Chinese Medicine, Guangzhou 510006, China.
[2]State Key Laboratory of Traditional Chinese Medicine Syndrome, School of Pharmaceutical Sciences, Guangzhou University of Chinese Medicine, Guangzhou 510006, China.
[3]Key Laboratory of Chronic Disease Prevention and Control of Traditional Chinese Medicine of Guangdong Higher Education Institutes, Guangzhou University of Chinese Medicine, KLGHEI (2024KSYS024), Guangzhou 510006, China.

[*] **Corresponding author:**
Prof. Tianqin Xiong, PhD
Pharmaceutical Building, Guangzhou University of Chinese Medicine, No. 232 Outer Ring Road, Panyu District, Guangzhou, Guang Dong 510006, China
Emile address: xiongtq020@gzucm.edu.cn

Prof. Changhui Liu, PhD
Pharmaceutical Building, Guangzhou University of Chinese Medicine, No. 232 Outer Ring Road, Panyu District, Guangzhou, Guang Dong 510006, China
Emile address: liuchanghui@gzucm.edu.cn

This PDF file includes:
Figures S1 to S6
Tables S1 to S3

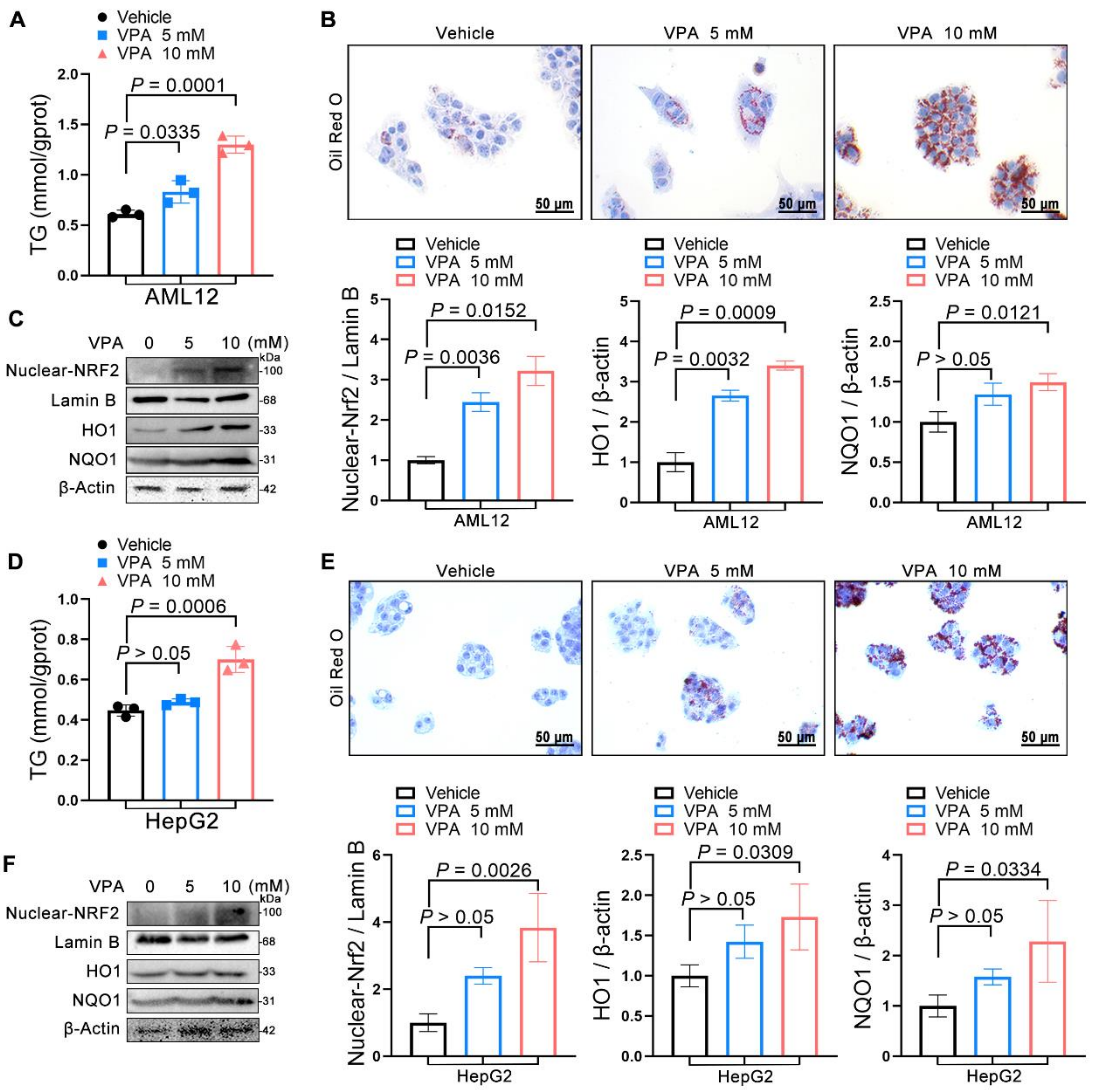

Figure S1. Correlations between NRF2 levels and the development of valproic acid-induced hepatic steatosis. (A) TG level of AML12 cells. **(B)** Oil red O staining of AML12 cells. Scale bar, 50 µm. **(C)** Protein expression of nuclear NRF2, HO1, and NQO1 in AML12 cells. **(D)** TG level of HepG2 cells. **(E)** Oil red O staining of HepG2 cells. Scale bar, 50 µm. **(F)** Protein expression of nuclear NRF2, HO1, and NQO1 in HepG2 cells. *n* = 3 biologically independent samples in (**A–F**). Statistical significance was determined using one-way analysis of variance. Data are presented as mean ± SEM.

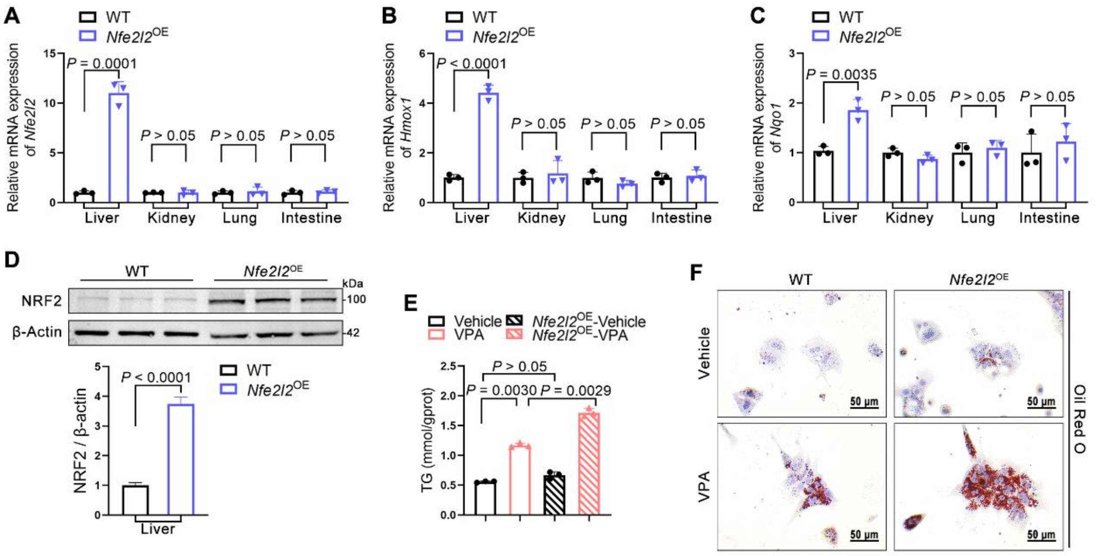

Figure S2. Evaluation of NRF2 overexpression efficiency and effects of NRF2 overexpression on VPA-induced hepatic steatosis. Mice in *Nfe2l2*^{OE} groups were obtained by injecting 5×10^{11} *v.g.* *Nfe2l2*-aav (diluted in PBS) into the tail vein of mice, whereas mice in WT group were administered equal amounts of vehicle solution. After completed 3–4 weeks of NRF2 overexpression, mice in WT and *Nfe2l2*^{OE} groups were dissected for the detection of NRF2 overexpression efficiency. **(A–C)** mRNA expression of *Nfe2l2*, *Hmox1*, and *Nqo1* in liver, kidney, lung and intestine. **(D)** Protein expression of NRF2 in liver. *n* = 3 mice per group in (**A–D**). **(E)** TG level of MPHs. **(F)** Oil red O staining of MPHs. Scale bar, 50 μm. *n* = 3 biologically independent samples in (**E, F**). Statistical significance was determined using one-way analysis of variance. Data are presented as mean ± SEM.

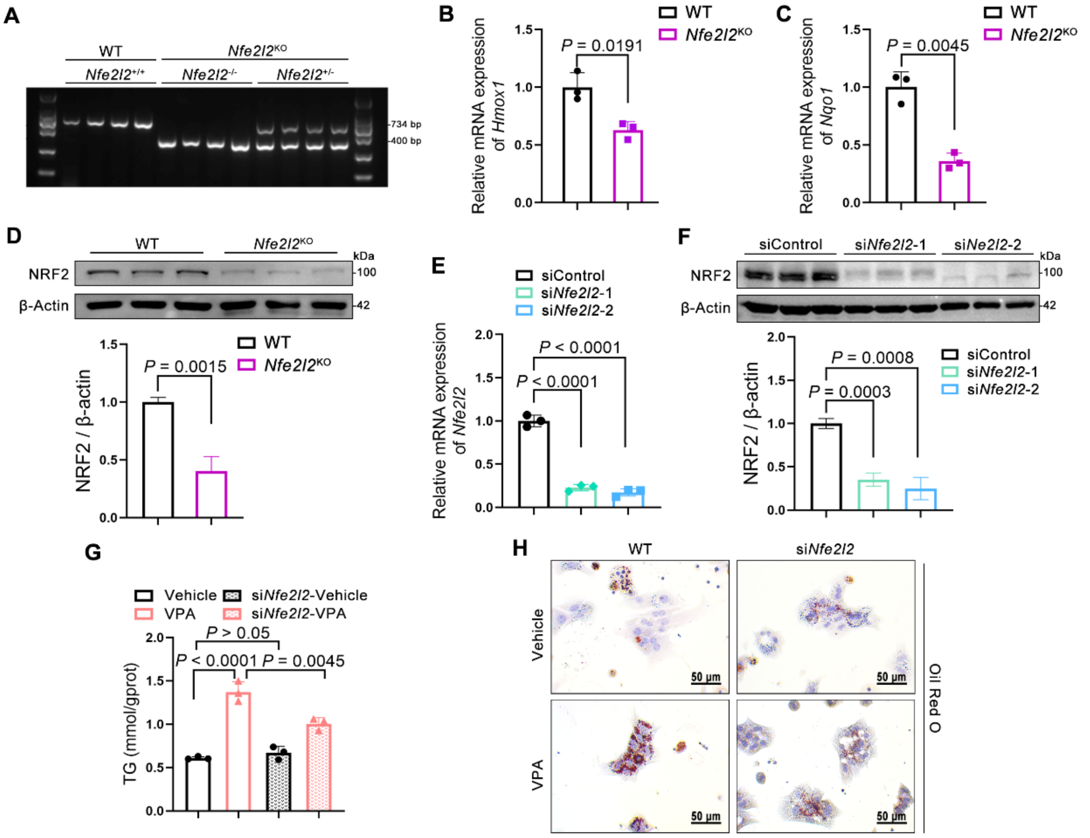

Figure S3. Evaluation of NRF2 knockout efficiency and effects of NRF2 knockout on VPA-induced hepatic steatosis. (A) DNA genotyping of mice in *Nfe2l2*$^{+/+}$, *Nfe2l2*$^{-/-}$ and *Nfe2l2*$^{+/-}$ group. $n = 4$ mice per group. *Nfe2l2* heterozygous mice (*Nfe2l2*$^{+/-}$) were used in all subsequent experiments because *Nfe2l2* homozygous mice (*Nfe2l2*$^{-/-}$) are highly susceptible to death by stimulation. **(B, C)** mRNA expression of *Hmox1* and *Nqo1* in liver. **(D)** Protein expression of NRF2 in liver. $n = 3$ mice per group in (**B–D**). **(E)** mRNA expression of *Nfe2l2* in MPHs. **(F)** Protein expression of NRF2 in MPHs. Evaluation of NRF2 knockdown efficiency after transfection of MPHs with si*Nfe2l2*-1 and si*Nfe2l2*-2. si*Nfe2l2*-2 was used in subsequent experiments due to its better knockdown effect compared with si*Nfe2l2*-1. **(G)** TG level of MPHs. **(H)** Oil red O staining of MPHs. Scale bar, 50 μm. $n = 3$ biologically independent samples in (**E–H**). Statistical significance was determined using one-way analysis of variance. Data are presented as mean ± SEM.

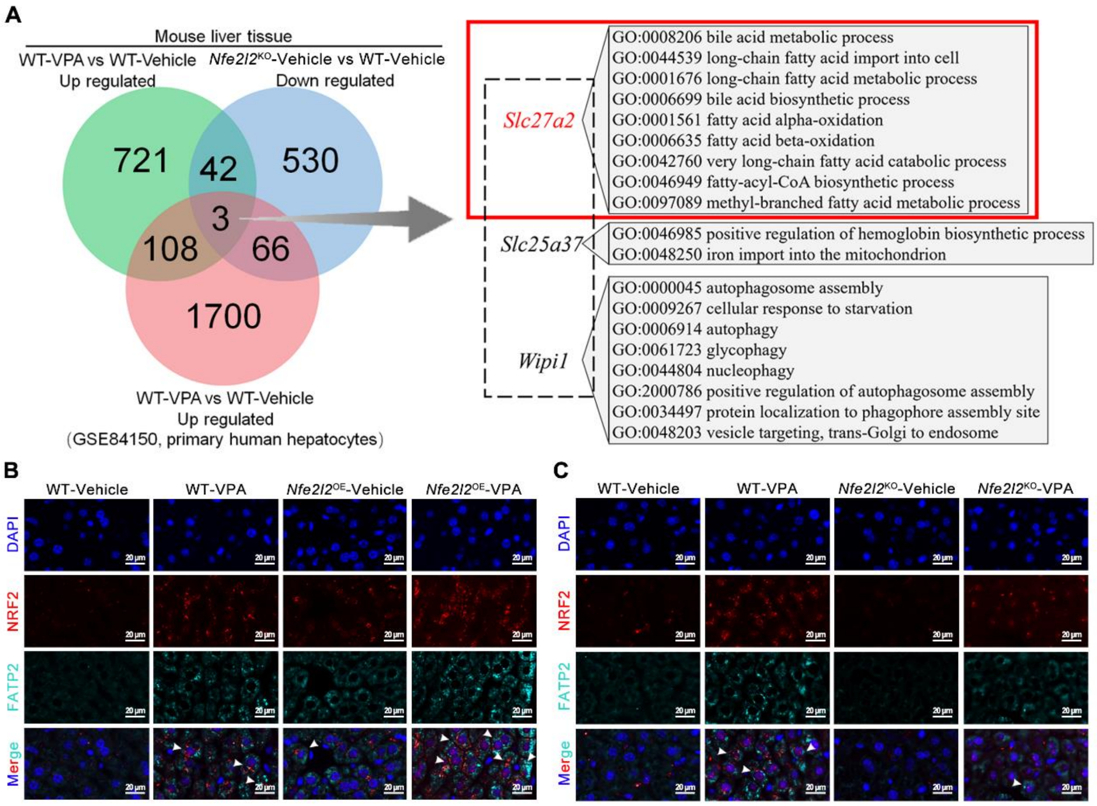

Figure S4. Identification of the downstream target of NRF2 that contributes to the progression of VPA-induced hepatic steatosis. (A) DEGs among upregulated genes from WT-VPA vs WT-Vehicle, downregulated genes from *Nfe2l2*^{KO}-Vehicle vs WT-Vehicle, and upregulated genes from WT-VPA vs WT-Vehicle in the GEO dataset and Gene Ontology analysis of DEGs. **(B)** Immunofluorescence staining of NRF2 (red) and FATP2 (turquoise) expression levels in liver from *Nfe2l2*^{OE} mice. Scale bar, 20 µm. **(C)** Immunofluorescence staining of NRF2 (red) and FATP2 (turquoise) expression levels in liver from *Nfe2l2*^{KO} mice. Scale bar, 20 µm.

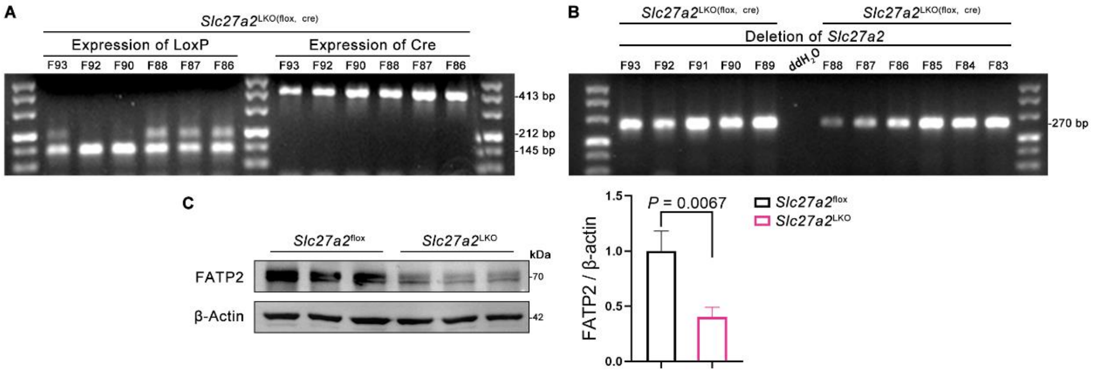

Figure S5. Evaluation of FATP2 knockout efficiency. (A, B) DNA genotyping of mice in *Slc27a2*$^{\text{LKO (flox, cre)}}$ group. (**A)** Determination of mice genotypes involving the LoxP and Cre. Flox (flanked lox) homozygous exhibit a band at 212 bp. Flox heterozygous exhibit bands at 212 bp and 145 bp. Cre-positive exhibit a band at 413 bp. n = 6 mice. **(B)** Evaluation of mice genotypes with *Slc27a2* deleted. Target gene deletion exhibits a band at 270 bp. n = 11 mice. **(C)** Protein expression of FATP2 in liver. n = 3 mice per group. Statistical significance was determined using one-way analysis of variance. Data are presented as mean ± SEM.

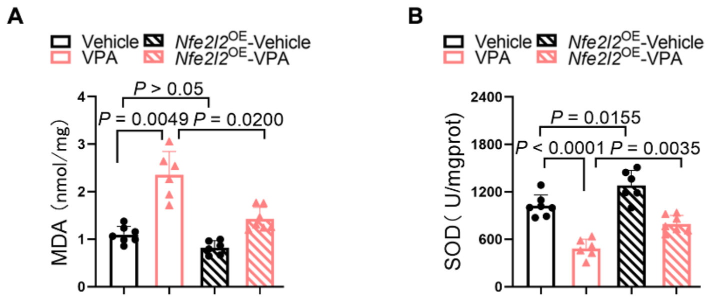

Figure S6. Oxidative stress levels on VPA-induced hepatic steatosis. (A) Levels of MDA in liver. **(B)** Levels of SOD in liver. WT-Vehicle group, *n* = 7 mice, WT-VPA group, *n* = 6 mice, *Nrf2*^{OE}-Vehicle group, *n* = 6 mice and *Nrf2*^{OE}-VPA group, *n* = 7 mice in (**A, B**). Statistical significance was determined using one-way analysis of variance. Data are presented as mean ± SEM.

Table S1. Molecular docking results of VPA on KEAP1.

Ligand	Binding site	Binding affinity (kcal/mol)
VPA	CYS288	-5.61
	ARG415	-5.36
	CYS151	-4.94
	CYS273	-4.70
	ARG483	-4.39

Table S2. The PCR primers sequence.

Species	Gene symbol	Sequence
Mus musculus	*Nfe2l2*	Forward: 5′-CAGCCATGACTGATTTAAGCAG-3′
		Reverse: 5′-CAGCTGCTTGTTTTCGGTATTA -3′
Mus musculus	*Hmox1*	Forward: 5′-TCCTTGTACCATATCTACACGG-3′
		Reverse: 5′-GAGACGCTTTACATAGTGCTGT-3′
Mus musculus	*Nqo1*	Forward: 5′-GAAGACATCATTCAACTACGCC-3′
		Reverse: 5′-GAGATGACTCGGAAGGATACTG-3′
Mus musculus	*Slc27a2*	Forward: 5′-CCCAGGATGTCATCTATACCAC-3′
		Reverse: 5′-CAATGTACTGAATGACCGTGAC-3′
Mus musculus	*Keap1*	Forward: 5′-TGCCCCTGTGGTCAAAGTG-3-3′
		Reverse: 5′-GGTTCGGTTACCGTCCTGC-3′
Mus musculus	*Gapdh*	Forward: 5′-GTTCCAGCACATTTTGCGAGT-3′
		Reverse: 5′-GGTGAGGTCGATGTCTGCTT-3′
Mus musculus	*LoxP*	Forward: 5′-AGCAGCTTGAACTAAAACTCTTGG-3′
		Reverse: 5′-TTGAAGACCCAGTAAAACGCTCTC-3′
Mus musculus	*Cre*	Forward: 5′-CATATTGGCAGAACGAAAACGC-3′
		Reverse: 5′-CCTGTTTCACTATCCAGGTTACGG-3′
Mus musculus	*Slc27a2*^{LKO}	Forward: 5′-AGCAGCTTGAACTAAAACTCTTGG-3′
		Reverse: 5′-CTCAACAAGGATACAGTTTGTGTG-3′
Homo sapiens	*SLC27A2*	Forward: 5′-AGCGGATTGAAGGCAGATGATGTC-3′
		Reverse: 5′-CGCAAGGCAAGAGTAGCACCAG-3′
Homo sapiens	*GAPDH*	Forward: 5′-AGAAGGCTGGGGCTCATTTG-3′
		Reverse: 5′-AGGGGCCATCCACAGTCTTC-3′

Table S3. Antibodies used in this work.

Antibody	Dilution	Source	Cat. No
NRF2	1:1000 for WB	Cell signaling technology	#12721
HO1	1:1000 for WB	Cell signaling technology	#43966
NQO1	1:500 for WB	Affinity	DF6437
FATP2	1:100 for WB, 1:50 for IF	Santa Cruz Biotechnology	sc-393906
KEAP1	1:1000 for WB	Proteintech	60027-1-Ig
Lamin B1	1:5000 for WB	Proteintech	12987-1-AP
β-Actin	1:20000 for WB	Proteintech	66009-1-Ig
p62	1:1000 for WB	Proteintech	31403-1-AP
p-p62	1:2000 for WB	Proteintech	29503-1-AP
LC3	1:1000 for WB	Proteintech	14600-1-AP
NRF2	1:100 for IF	Affinity	AF0639
Goat anti-rabbit IgG	1:3000 for WB	Proteintech	RGAR001
Goat anti-mouse IgG	1:3000 for WB	Proteintech	RGAM001
CoraLite594-conjugated goat anti-rabbit IgG	1:100 for IF	Proteintech	SA00013-4
CoraLite488-conjugated goat anti-mouse IgG	1:100 for IF	Proteintech	SA00013-1
